# Evaluation and implementation of QR Code Identity Tag system for Healthcare in Turkey

**DOI:** 10.1186/s40064-016-3020-9

**Published:** 2016-08-30

**Authors:** Vassilya Uzun, Sami Bilgin

**Affiliations:** Alanya HEP University, Alanya, Turkey

**Keywords:** Medical informatics, Patient identification, Personal health records, Turkey

## Abstract

For this study, we designed a QR Code Identity Tag system to integrate into the Turkish healthcare system. This system provides QR code-based medical identification alerts and an in-hospital patient identification system. Every member of the medical system is assigned a unique QR Code Tag; to facilitate medical identification alerts, the QR Code Identity Tag can be worn as a bracelet or necklace or carried as an ID card. Patients must always possess the QR Code Identity bracelets within hospital grounds. These QR code bracelets link to the QR Code Identity website, where detailed information is stored; a smartphone or standalone QR code scanner can be used to scan the code. The design of this system allows authorized personnel (e.g., paramedics, firefighters, or police) to access more detailed patient information than the average smartphone user: emergency service professionals are authorized to access patient medical histories to improve the accuracy of medical treatment. In Istanbul, we tested the self-designed system with 174 participants. To analyze the QR Code Identity Tag system’s usability, the participants completed the System Usability Scale questionnaire after using the system.

## Background

In recent years, Turkish citizens’ level of health has clearly increased. The Health Transformation Program reform platform, with its motto of “People First,” has been a major contributor to this successful improvement. The Turkish government is now putting the second phase of this program into action to enhance governance, efficiency, and quality in the health sector. As part of the health reforms, legal changes have been made within the Ministry of Health (MoH), such as restructuring and reorganization of its units and affiliates. The restructuring efforts strive to empower the MoH’s management function and improve its health system policy development, planning, supervision, monitoring and evaluation. To reinforce the MoH’s position in healthcare service provision, the Public Health Institution has been created (World Health Organization 2006; Savas et al. 2002).

According to a report by the Organization for Economic Cooperation and Development (OECD), Turkish citizens’ level of satisfaction is increasing (Ada et al. 2013). However, much work remains to be done to improve their satisfaction with healthcare (Camgöz-Akdag and Zineldin 2010; Yasar 2011; Kacak et al. 2014).

Therefore, we have developed a patient identification system that could be integrated into the developing Turkish healthcare system. When used by healthcare personnel or police, this system would identify and provide essential information about the patient and decrease medical errors, which in turn would increase Turkish citizens’ satisfaction with healthcare. Although systems have been successfully implemented in various countries, one has not been implemented in Turkey; thus, that aspect of our system is novel.

Because any failure or error throughout the patient identification process might lead to irreversible consequences, patient identification is important throughout the course of medical service provision. Such errors can be prevented by professionals taking low-cost precautions defined in institutional protocols (Bates et al. 2009).

In 2007, the World Health Organization (WHO), in partnership with Joint Commission International (an American accrediting agency), released nine patient safety solutions to prevent errors and adverse healthcare events. These solutions consist of system projects or interventions that can prevent or attenuate patient harm: managing the risks associated with lookalike/sound-alike medication names; correct patient identification; handoff communication during transfer of patient responsibility; performance of the correct procedure on the correct body part; control of concentrated electrolyte solutions; administration of appropriate medication throughout care; avoidance of catheter and tubing misconnections; needle reuse and injection device safety; and improved hand hygiene to prevent healthcare-associated infections (World Health Organization 2007, 2008).

Patient identification is widely seen as addressable by solutions and as a necessary and vital element of healthcare and patient safety. When patient identification instructions are followed, the underlying methods are capable of preventing many errors and undesired consequences at various steps of healthcare provision.^1^

Patient identification is still performed manually by many public healthcare providers. This method of patient identification should be replaced, because it is very likely to lead to anthropogenic errors. Identification errors can lead to severe problems with medical procedures, such as medication administration, blood transfusions, clinical trials, and surgery (García-Betances and Huerta 2012).

One method to minimize these risks is through the use of automated patient identification systems to identify patients (García-Betances and Huerta 2012; Frisch et al. 2009). These systems quickly and reliably identify patients, besides facilitating ease of access and management of patient medical histories (i.e., personal health records; PHR). The main objective of these systems’ design is to guarantee the reliability and security of patient identification and speed of access to clinical information (Tang et al. 2006; Kahn et al. 2009).

Besides many healthcare technology companies, several research groups have also designed automated patient identification systems. Two widely used technologies employ graphical one-dimensional (1D) codes (Ebling and Cáceres 2010) printed on labels, widely known as barcodes, or radio frequency identification tags (RFID) (Want 2006). In automated patient identification practice, the former are the technologies used most by healthcare centers (Turner et al. 2003; Patterson et al. 2002; Poon et al. 2010; Sandler et al. 2004; Morrison et al. 2010; Murphy and Kay 2004; Hayden et al. 2008; Pagliaro and Rebulla 2006; Thuemmler et al. 2007; Dzik 2007; Halamka 2005; Booth et al. 2006; Perrin and Simpson 2003), but different technologies also exist. Many such technologies rely on modification of barcodes into two-dimensional (2D) graphical codes (García-Betances and Huerta 2012; Tang et al. 2006). These new 2D codes can be considered as an important improvement on barcodes, because although they can be generated and printed equally easily to 1D codes, they can store greater amounts information. Furthermore, these 2D codes have extra features, such as error correction (Soon 2008).

Although there are many options for the 2D code used to identify patients, the QR code (Denso 2011) technology is a practical one: the healthcare industry is seeing widening use of applications relying on QR codes (Vazquez-Briseno et al. 2010; Liu et al. 2012; Al-Khalifa 2008; Gómez et al. 2013; Rahman et al. 2015; Leza et al. 2013a, b; Emran and Leza 2014; Avidan et al. 2015; Bellot et al. 2015; Shakil et al. 2014; Shejul et al. 2015; Dube et al. 2015; Charoensiriwath et al. 2015; Lee et al. 2015). The most significant advantage of QR code utilization for patient identification originates from its simple technological base: QR code-based technology provides high ease of access for users, because it requires no special tags (such as RF tags). QR codes are created very easily, and because they can be printed on any surface (e.g., paper or plastic labels or any other surface), they do not require any equipment more specialized than a printer (García-Betances and Huerta 2012; Soon 2008; Denso 2011).

Because smartphones are widely used across various life domains, reading and decoding QR codes has become much easier than using systems based on complex technology. A QR code system has another advantage over RFID-based systems: because reading QR codes requires closer proximity, it is almost impossible to read an undesired code. From this perspective, QR code reading is unambiguous, as it only requires close proximity of the reader device to the patient’s bracelet to read the code (García-Betances and Huerta 2012; Soon 2008; Denso 2011).

QR code-based technology is also superior in other ways, such as higher data storage capacity, lower implementation cost, technical simplicity, widespread use, and widely available, free programs for reading and decoding by camera-equipped smartphones. These features make this technology attractive for patient identification purposes, especially for institutions in developing countries with limited sources (García-Betances and Huerta 2012). Turkey is a developing country with a limited budget: its total health spending accounted for 5.4 % of GDP in 2012, the lowest share among OECD countries and well below the OECD average of 9.3 % (OECD Health Statistics 2014).^2^ Therefore, the system has to be cost-efficient and easily implemented. These requirements make QR code technology the best choice for our system, which will be used for both QR code-based medical identification alerts and patient identification in hospitals.

Medical identification alert systems can provide first responders or emergency personnel with immediate access to the PHR (i.e., health records containing information related to patient care maintained by the patient) of a person in distress. PHRs are usually maintained by web services; thus, there is no theoretical limit on their amount of data storage (Tang et al. 2006; Kahn et al. 2009). Solutions including HealthVault^3^ and PatientsLikeMe^4^ allow for data to be shared with other applications or specific people.

We are interested in QR code-based solutions. QR code-based medical identification alerts are provided by scanning QR code-equipped medical alert stickers placed on items such as bracelets, necklaces, or ID cards. Patient emergency information is accessed through the QR code’s embedded web link. Emergency personnel access the emergency information by scanning the QR code with a smartphone. Many QR code-based medical identification alert services are available worldwide (Patterson et al. 2014; Langford and McCoy 2012; Mitsunaga 2012; Bennett 2012; Walton 2015; Tregnaghi 2013; Fire department tests QR codes for citizen medical data 2012),^5^ such as ICE ID,^6^ ENDEVR,^7^ If I Need Help,^8^ Keep Me Safe ID,^9^ LIFETAG,^10^ TAGGISAR,^11^ and Elegant Medical Alert.^12^ However, most of those are paid services and not part of any national healthcare system. The system we propose will be freely available to all Turkish citizens as a service provided by the Turkish healthcare system.

Similarly, in Marin County, California, a pilot study has been conducted to use QR codes as a tool for identifying patients through patient information, such as medical history and allergies. Those data are embedded directly within the QR code and can be accessed easily by scanning the code when needed (Fire department tests QR codes for citizen medical data 2012).

ICEid (see footnote 6) developed ICEid Tags and ICEid QR CODE stickers to identify patients and provide their medical information in case of emergency. ICEid Tags are plastic and waterproof; they can be machine washed while attached to clothes or attached to zippers during cycling, running, or any other outdoor activity. They can also be attached to school bags, handbags, and backpacks. The ICEid QR CODE sticker can also be attached to a bicycle, motorbike, scooter, or any helmet. Both ICEid Tags and ICEid QR CODE stickers feature unique QR codes and matching alphanumeric codes, which can be scanned to access personal and medical information; alternatively, this information can be accessed via the ICEid website.

childIDcode (see footnote 5) has developed identification stickers for children prone to wandering and elopement; these waterproof stickers are custom-printed with QR codes. When the code is scanned with a smartphone, it will display, for example, “I Have Autism,” along with an emergency contact. The stickers can be placed on articles of clothing such as hats or backpacks, and they are useful for school field trips, vacations, or even daily outings where a child may be at risk of wandering. They are also a good option for children with sensory issues who are unable to tolerate identification bracelets.

If I Need Help (see footnote 8) is a nonprofit organization that works to reunite those who are lost, disoriented, or cannot self-advocate with their loved ones and caregivers. They use QR code patches for emergency identification. These QR code patches can be sewn onto clothing, or a variety of clothing may be purchased with the QR code patches already attached.

QR codes have also been widely used for patient identification in hospital environments. This technology is in use in many medical facilities, mainly in the Asia–Pacific region, for patient processing and access to and control of patient data. Healthcare centers in Japan, Singapore, and Hong Kong have established the Unique Patient Identification system, which transitioned from barcodes to QR codes in 2008 (Soon 2008; HA Quality and Risk Management Annual Report^2010^).

Brenmoor^13^ has developed a variety of hospital bracelets and blood bag tags that employ QR codes.

Addenbrooke’s Hospital in Cambridge also utilizes QR codes in accordance with its patient safety policy. The QR code is printed next to the other personal information on a bracelet worn by the patient. Initially, the system was used only to track blood transfusions and control the coincidence between patient and blood type. However, the QR codes are now used much more widely to minimize all types of medical failures (Solutions 2015).

An update to the KBMA 1D code-based identification system at Houston’s Methodist Hospital has been proposed, whereby its old barcode-based patient identification will be migrated to a QR code-based system (General Data Company 2008).

In (Charoensiriwath et al. 2015), the authors implemented the use of QR Codes to identify patients in a hospital. This information is used in a mobile application used by hospital staff to register patient-related activities.

A Taiwanese study group has tested a pilot program wherein QR codes are used for patient identification and computerized transfer of prescription information between the hospital and pharmacies. The QR code technology was utilized successfully and provided satisfactory results (Lin et al. 2012).

Two Venezuelan researchers performed an investigation of patient identification technologies and determined that QR codes had optimal features for storage of large amounts of data, especially at institutions or projects with low budgets (García-Betances and Huerta 2012).

We have developed a QR Code Identity Tag system to be integrated into the Turkish healthcare system. It consists of a QR code-based medical identification alert system and a patient identification system for hospital environments. A unique QR code tag is generated for each member of the medical identification alert system and each patient. When used to facilitate medical identification alerts, the QR Code Identity Tag can be placed on a bracelet, necklace or identification card, whereas while on hospital grounds, patients need to wear QR Code Identity bracelets. The QR codes contain links to the QR Code Identity web page, which contains detailed information about the QR code holder. The code is scanned and decoded using the smartphone application or any QR code scanner. This application provides hierarchical levels of privacy, so that emergency medical responders (e.g., paramedics, firefighters, or police) can access more details than the average smartphone user can. Emergency medical responders and medical care providers can instantly retrieve a patient’s medical history to provide more informed medical care.

We tested the developed system on a set of 174 volunteers from various areas of Istanbul. To evaluate system usability, after using the QR Code Identity Tag system, volunteers were asked to complete the System Usability Scale (SUS) (Brooke 2013; Brooke et al. 1996) questionnaire. Many studies have been conducted to assist practitioners in assessing product/service usability. While many studies have focused on specific interface types, information from other studies may be used to analyze a wider range of interface types. In our study, we used the SUS questionnaire because it has the capacity to analyze a wide range of products and services with verified validity. Some features of the SUS make it quite useful. First, it is simple, consisting of only 10 statements, and is quick and easy for subjects to complete. Second, it is open source, free to use, and quick to score. Third, it employs agnostic technology, meaning that it can be used to analyze various types of user interfaces, such as websites, cell phones, interactive voice response systems (both touch-tone and speech), and TV applications. Finally, the result of the survey is a single score that varies between 0 and 100. Thus, it is relatively easy to understand for researchers from other disciplines who work on project teams (Bangor et al. 2008; Lewis and Sauro 2009; Sauro 2011; Bangor et al. 2009; Tullis and Stetson 2004).

We evaluated all the questionnaires completed by the participants and obtained the mean SUS score, which is above the success threshold; thus, the developed system is usable and satisfactory.

## QR Code Identity Tag

At the scene of a medical emergency or in a hospital environment, the QR Identity Tag allows medical personnel, emergency medical technicians (EMTs), police, or firefighters to identify the person and instantly obtain his/her vital information.

The QR Code Identity Tag allows members to control their own PHRs by possessing or editing the information. Each person is assigned a unique QR code tag, which can be printed through the QR Identity Tag website or by medical personnel during hospital registration. The QR Identity Tag can be placed on any object: a necklace, bracelet, keychain, identification card, or tattoo.

**Fig. 1:**
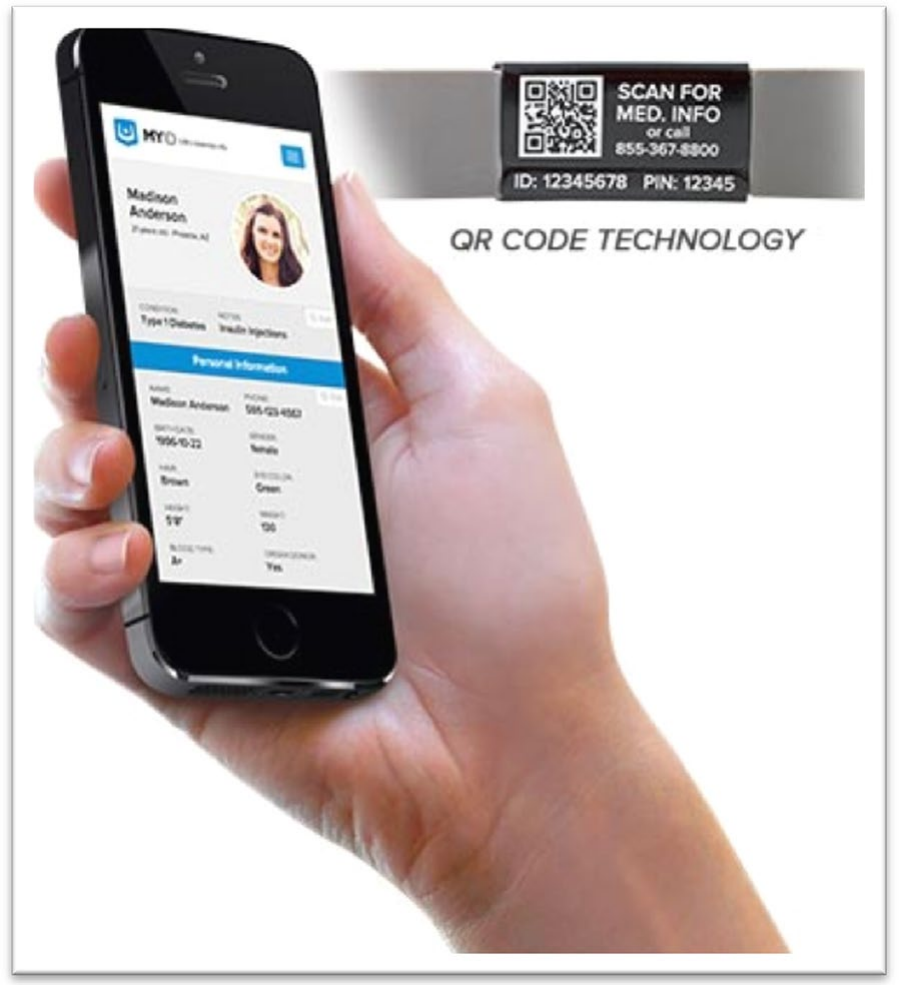
Smartphone and QR identity bracelet

When the QR code is scanned by medical personnel, EMTs, police, or firefighters using the QR Identity Tag application on their smartphones, detailed patient information is displayed, as illustrated in Fig. 1.

We prefer to employ QR code-based identification, because QR code-based tag technology, when read and decoded by smartphones, is considered the most useful and cost-efficient alternative for automatized patient identification. Further, it provides quick remote access to health records by medical professionals in public healthcare systems with limited budgets.

### Patient identification in hospital environment

To receive treatment, each patient first needs to register with the hospital system. A unique online profile, password, identification number, and QR code are generated for each patient, and a bracelet containing the QR code is placed on the patient’s wrist. Incoming patients are also required to provide a detailed medical history. In most hospitals, patients complete a paper copy of the form; however, in our system, patients are encouraged to use on online form, which can be accessed by scanning the QR code on the identity bracelet. The form includes an emergency contact phone number, address, telephone number, blood type, contact information of a person with the same blood type, allergies, illnesses, special conditions, current medications, and other information.

Several copies of the QR code are produced for each patient admitted into the hospital, and they are used wherever patient identification is required. Copies of the QR code are placed on the door of the patient’s room, the bed, and the chart so that medical personnel can quickly identify the patient and obtain his/her medical history and treatment status. The QR codes are scanned by doctors before surgeries to identify the patient and avoid surgical errors. QR codes are also placed on the patient’s lab samples to prevent errors and maintain information privacy.

Patient misidentification in hospitals is a very serious problem. To reduce its risk of occurrence, we suggest that patients wear two bracelets, one on each hand, or a necklace; that would be useful in a scenario when one of them is damaged or lost. Double identification is also useful in cases where the wrong QR code was printed during registration. One QR code is printed by the patient and placed on a bracelet, and the second QR code is printed by the hospital and placed on a bracelet on the other hand. This is done in case a hospital clerk makes a mistake and prints a QR code belonging to another patient. After printing the two QR codes, medical personnel should scan both of them to make sure that they belong to the same person.

### Outpatient identification

Some patients may always wear the QR code identity bracelet/necklace/identification card, which could be useful for patients with, e.g., allergies, asthma, unusual blood types, HIV/AIDS, diabetes, and Alzheimer’s.

Initially, interested parties need to register themselves or those under their care with the QR Code Identity Tag website. The user is asked to choose a strong password. After the account is created, the member is prompted to enter detailed information, including an emergency contact phone number, addresses, telephone numbers, blood type, contact information of a person with the same blood type, allergies, illnesses, special conditions, and current medications.

When the process is completed, a security code and a QR Code Identity Tag are generated for the user, and the user is advised to share the Security Code with the listed emergency contact person. The QR Code Identity Tag can be printed, or the member can order a bracelet, necklace, or keychain encoded with it. Users can change their information anytime by logging in into the web system. The QR Code Identity Tag does not have to be reissued, as it contains not information but a link to the password-protected web page.

### Authorization

Medical personnel, EMTs, police, or firefighters may install the QR Code Identity Tag mobile application and log into the system to gain easy access to the information. They are assigned privileges to obtain the information without contacting the next of kin for rapid access during an emergency.

When an authorized individual listed above scans the QR Code Identity Tag, they obtain detailed information about the person to whom the tag belongs directly. When someone without authority scans the tag, contact information of the emergency contact is displayed. In a hospital environment, both the patient’s personal details and medical records are available to medical personnel.

## System design

**Fig. 2:**
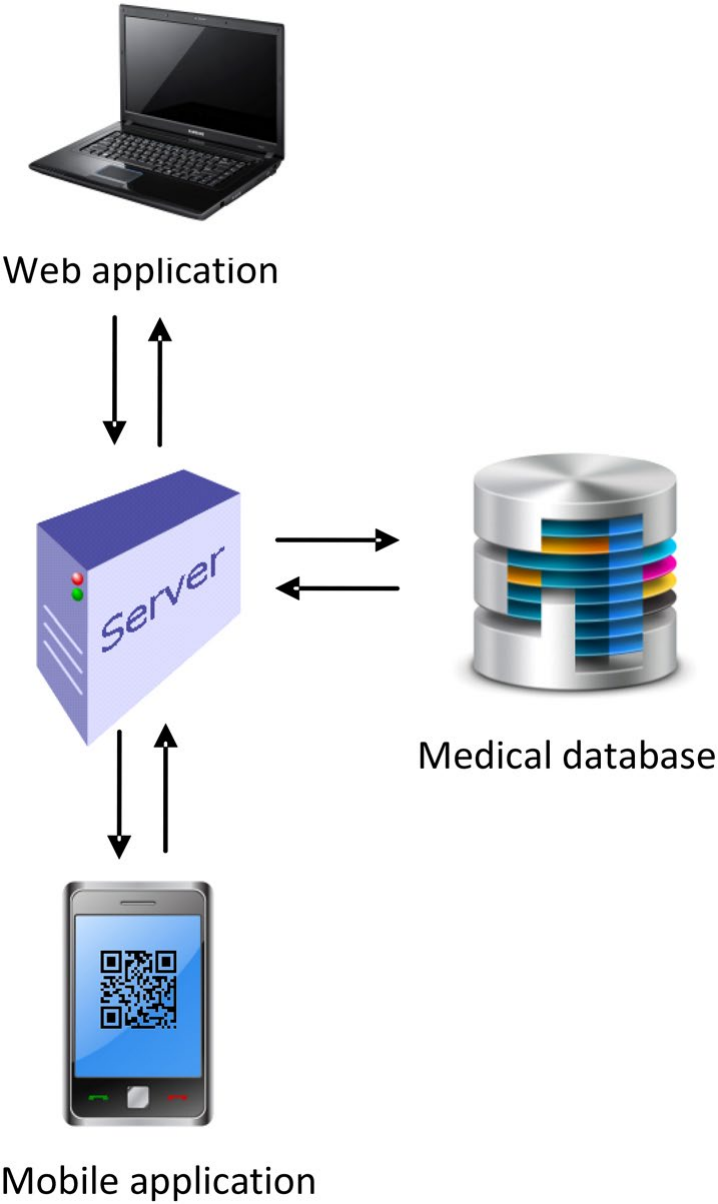
System architecture

Our system consists of three parts: website, database, and mobile application (Fig. 2). The website is used to maintain the PHRs stored in the database, while the mobile application provides easy access to them.

### Information access

**Fig. 3:**
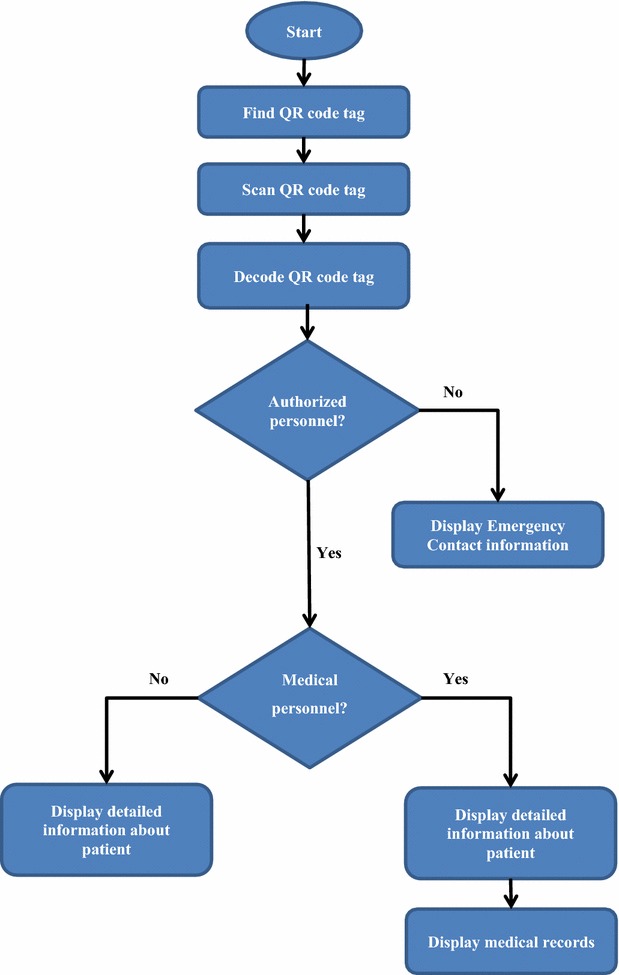
Information access

Emergency contact information can be obtained by scanning the code with any standard QR Code reader, but additional information is retrieved and revealed when the code is read with QR Code Identity Tag mobile application. This helps emergency medical responders to provide more informed medical care. The additional information, potentially in the form of PHRs, can be retrieved with authentication. Figure 3 shows how a QR code is scanned and how basic or detailed information is accessed.

### Emergency responders’ authentication

A proper authentication paradigm assures that the correct individuals have access to medical records occurs in the correct contexts. Authorized personnel include police, firefighters, EMTs and hospital medical personnel, to whom the application will be distributed freely by the Turkish healthcare system. The application is downloaded from protected websites, and login to the system proceeds using privately assigned usernames and passwords. When medical personnel scan the QR code using the mobile application in a hospital environment, in addition to detailed patient information, they can access the patient’s medical records. However, when other authorized personnel scan the code, they can only access the detailed information about the patient.

### Software tools

The Adobe CS6^14^ design package was used to design the website, and the Dreamweaver software package was used to combine these designs using the HTML and PHP coding languages.

Although PHP is a server-side scripting language that was designed for web development, it is also a general-purpose programming language with thousands of functions that handle almost any task easily.

A MySQL^15^ database was used to keep the medical records. MySQL is the world’s second most widely used open-source relational database management system (RDBMS). For design of applications intended to operate on any device, regardless of operating system, we prefer to use PHP and MySQL, because it eases programming tasks.

The MySQL database system can be used on the web but runs on a server. It is a fast, easy-to-use RDBMS used by businesses of all sizes. This database system is compatible with many operating systems and works with many languages, including PHP, Perl, C, C++, and Java. MySQL uses a standard form of the well-known SQL data language to store, organize, and retrieve data.

We developed a mobile application for Android^16^ smartphones. The Android operating system is based on the Linux kernel, and its user interface operates via direct manipulation, its primary targets being touchscreen mobile devices such as smartphones and tablets. We prefer to use Android, because as of 2011, it has the largest install base of any mobile OS, and as of 2013, more Android devices were being sold than the sum of Windows, iOS, and Mac OS devices. Besides Android, we also plan to develop applications for other mobile operating systems.

The Android application was developed using Eclipse^17^ to implement the Android Java programming language. Eclipse is an integrated development environment (IDE) that contains a base workspace and an extensible plugin system for environmental customization. Written mostly in Java, Eclipse can be used to develop applications.

The Android software development kit (SDK)^18^ was used to create the new application for the Android operating system; it includes a comprehensive set of development tools, such as a debugger, libraries, a QEMU-based handset emulator, documentation, sample code, and tutorials. Although the officially supported IDE is Eclipse using the Android Development Tools (ADT) plugin, all editions of IntelliJ IDEA IDE^19^ also fully support Android development out of the box. The proposed Android application’s client-side functionality was developed in Eclipse and integrated with Android SDK.

The NetBeans IDE was written in Java and can run anywhere a Java Virtual Machine is installed, including Windows, Mac OS, Linux, and Solaris. A JDK is necessary for Java development functionality but is not required for development in other programming languages. The application’s server-side functionality was built in the NetBeans IDE environment.

### Database design and connections

**Fig. 4:**
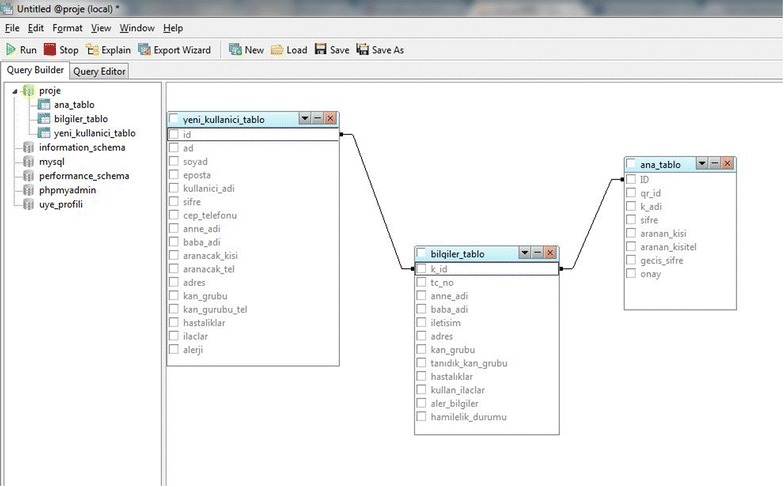
Database design

We used MySQL database to store the PHRs. During database design, one of our objectives was to store more data in fewer tables. Here, the data entered by the user during signup are recorded in table yeni_kullanıcı_tablo, and in table bilgiler_tablo, these data are recorded and assigned roles. In table ana_tablo, the application controls the password seen on the screen during initial scanning. The database design is depicted in Fig. 4.

**Fig. 5:**
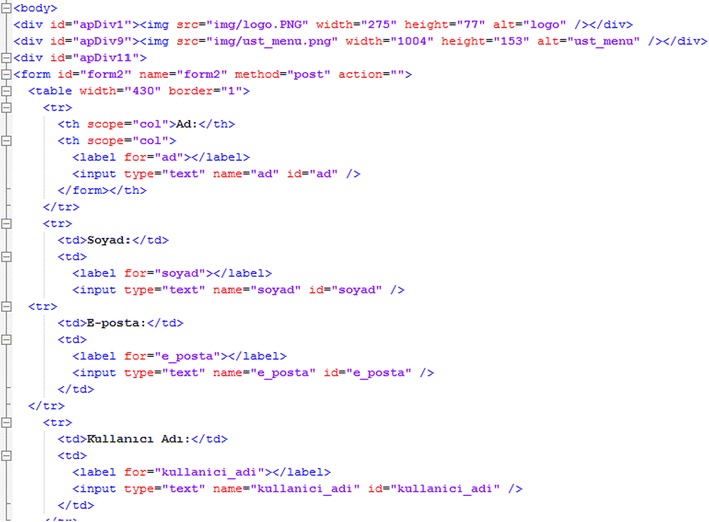
Code of the main webpage

Our website has a simple, user-friendly design. The code sections are constructed according to a systematic structure (Fig. 5).

**Fig. 6:**
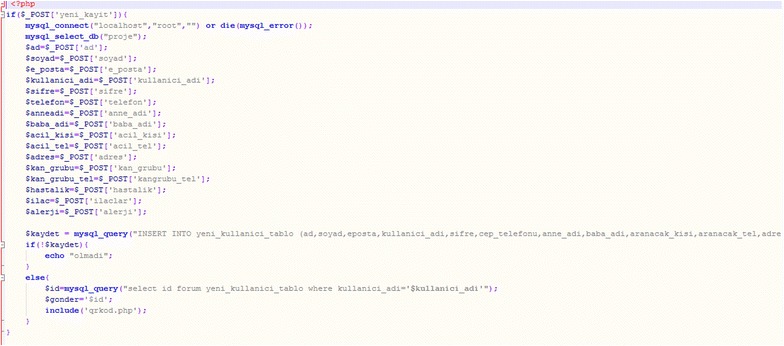
Code for registration of new users

In our webpages’ code, we establish the database connection in first line and make the required comparisons. First, the codes used for registration of new users are seen below (Fig. 6).

**Fig. 7:**
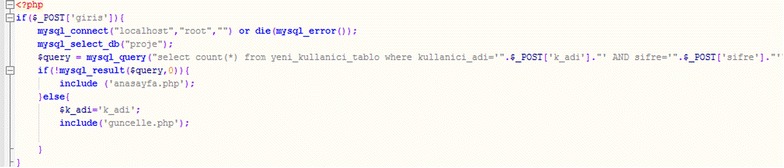
Code for the password check

Then, to match the username and password, the web database is connected, and the password is checked (Fig. 7).

**Fig. 8:**
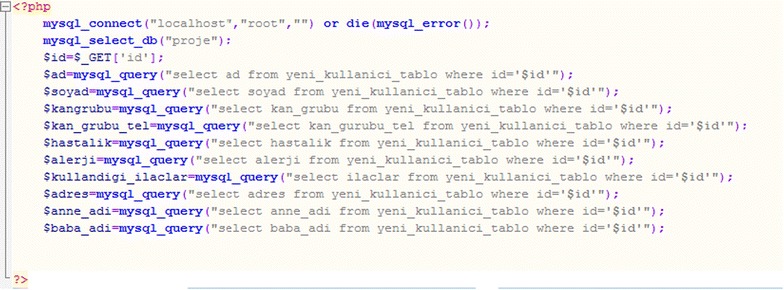
Code for obtaining user information from the database

Then, after login is complete, the incoming user’s identification is taken. The information in the database’s tables is shown in Fig. 8.

### Screenshots of the system

**Fig. 9:**
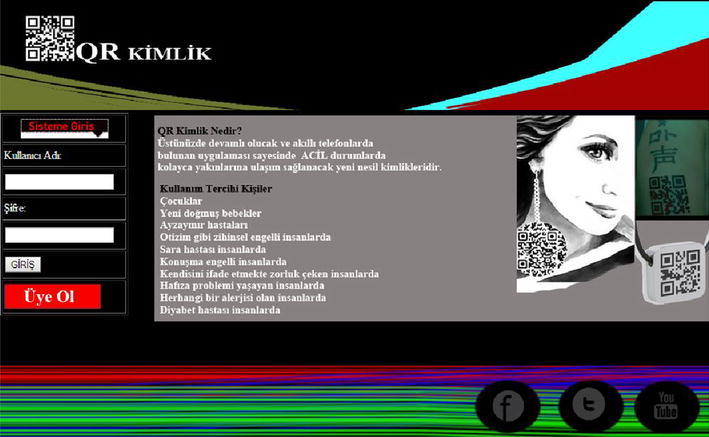
Screenshot of the main web page

**Fig. 10:**
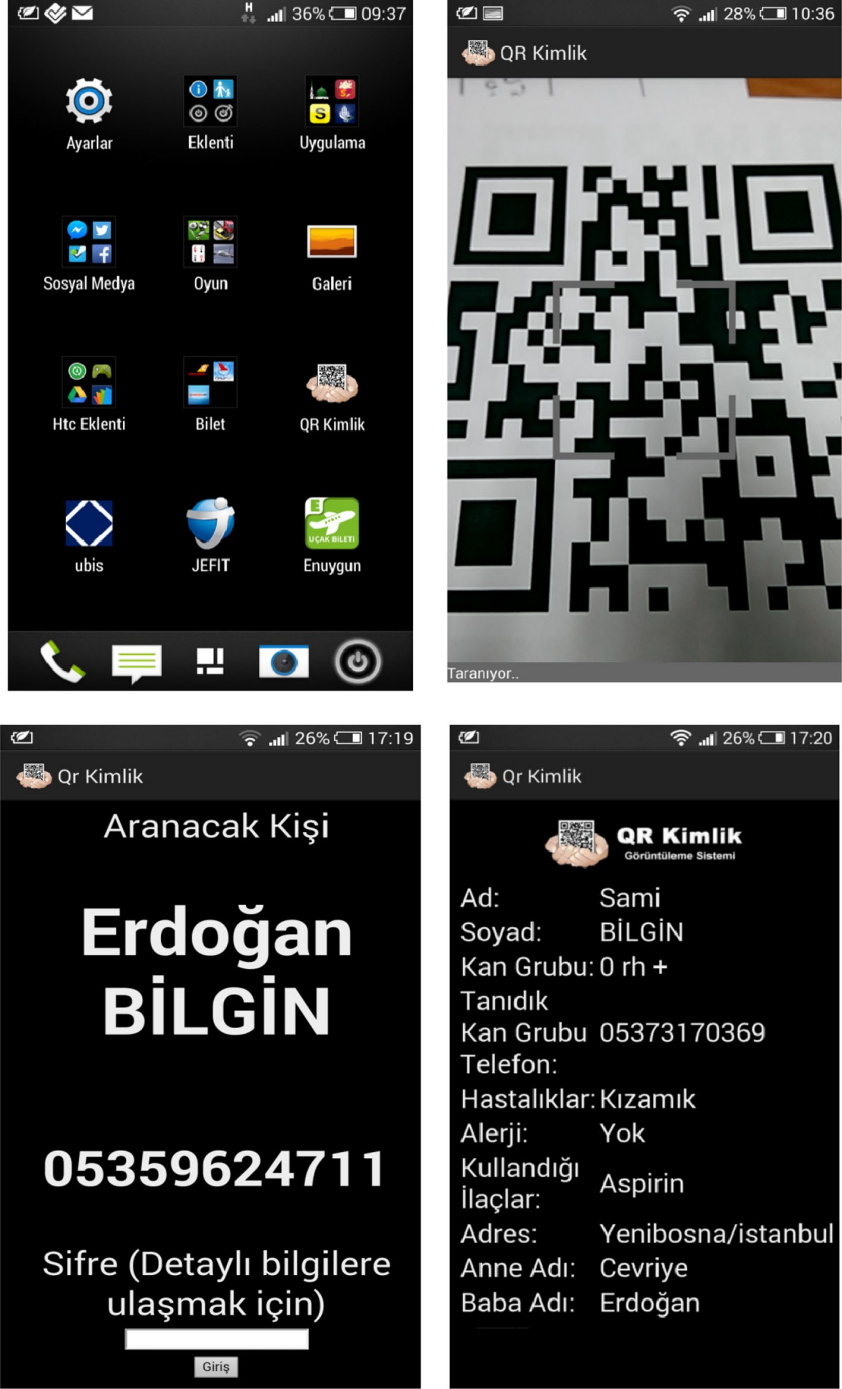
Screenshots of the mobile application

The following are some screenshots of the QR Identity Tag system (Figs. 9, 10).

## Performance evaluation

We evaluated the performance of the system using the Brooks’ SUS (Brooke 2013; Brooke et al. 1996) questionnaire, a simple, 10-item attitude Likert scale that covers a variety of aspects of system usability, such as need for support, training, and complexity (Bangor et al. 2008; Lewis and Sauro 2009; Sauro 2011; Bangor et al. 2009; Tullis and Stetson 2004).

Sauro (2011) stated that a translated version of the SUS had similar internal reliability to the original English version. Because our system was developed in Turkish and for Turkish citizens, we translated the questionnaire into Turkish.

### SUS questionnaire

The question items included in the SUS survey were judged according to a five-point Likert scale ranging from strongly disagree (1) to strongly agree (5):

1. I think that I would like to use this system frequently.
2. I found the system unnecessarily complex.
3. I thought the system was easy to use.
4. I think that I would need the support of a technical person to be able to use this system.
5. I found the various functions in this system well integrated.
6. I thought there was too much inconsistency in this system.
7. I would imagine that most people would learn to use this system very quickly.
8. I found the system very cumbersome to use.
9. I felt very confident using the system.
10. I needed to learn a lot of things before I could get going with this system.

While the SUS was only intended to measure perceived ease of use (i.e., a single dimension), recent research has shown that it provides a global measure of system satisfaction and subscales of usability and learnability. Items 4 and 10 provide the learnability dimension, and the other eight items provide the usability dimension; both subscales and the global SUS score can thereby be tracked (Sauro 2011).

### SUS calculation

To rescale all values as 0–4, 1 was subtracted from the participant’s response for odd-numbered items, while for even-numbered questions, the participant’s response was subtracted from 5. The converted responses were summed for each user, and the total was multiplied by 2.5 to convert the range of possible values from 0–40 to 0–100 (Sauro 2011).

The overall SUS score success threshold (i.e., the minimum acceptable point at which users are likely to recommend the application to a friend) is 80.30. If the overall mean SUS score is above the success threshold, then the application is considered satisfactory and usable overall (Sauro 2011).

### Process

A usability evaluation was conducted on three groups of volunteers. After performing the assigned tasks, each participant was asked to complete a SUS questionnaire. After completing the survey, each participant was also asked whether the QR Code Identity Tag system should be integrated into the Turkish healthcare system.

## SUS results

### Group A

The first group consisted of 54 volunteers of various demographics. There were 18 volunteers in each of the age ranges of 18–30, 31–50, and 51–70 years. In total, 27 female and 27 male volunteers participated in the study. Of the participants, 27 had no computer experience, and 27 used computers daily for such activities as email, word processing, and internet use. In each group, nine participants were requested to wear each of the QR Code Identity necklace, QR Code Identity bracelet, and QR Code Identity card for 1 week.

Each participant was requested to perform the following tasks:

1. Register on the QR Code Identity Tag website
2. Enter detailed information on the QR Code Identity Tag website
3. Update detailed information on the QR Code Identity Tag website
4. Scan the QR code
5. Wear the QR Code Identity necklace/bracelet/identification card.

**Fig. 11:**
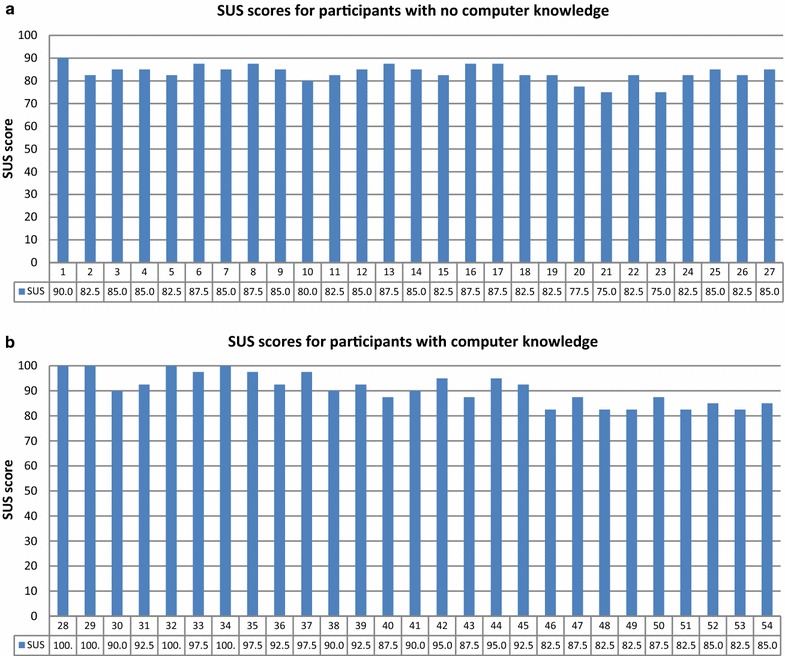
SUS score distribution for each participant **a** with no computer knowledge and **b** with computer knowledge

First, we evaluate performance according to the participants’ computer knowledge. The SUS score distributions for each participant are separated by computer knowledge level in Fig. 11.

**Fig. 12:**
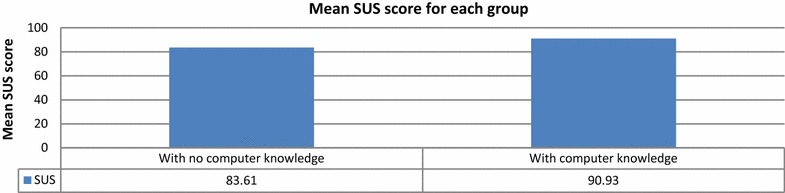
Mean SUS scores for groups with and without computer knowledge

Figure 12 depicts the mean SUS scores for the groups with and without prior computer knowledge. The mean SUS score for the group without computer knowledge (83.61) is much lower than that for the group with computer knowledge (90.93). Because the first group had no computer experience, they sometimes needed technical support to be able to use the system, and they felt that they needed to learn things before they could get going with the system. However, the group with computer experience felt competent using the system. Although the participants with no computer knowledge had some difficulties using the system, both groups’ mean SUS scores were above the success threshold.

**Fig. 13:**
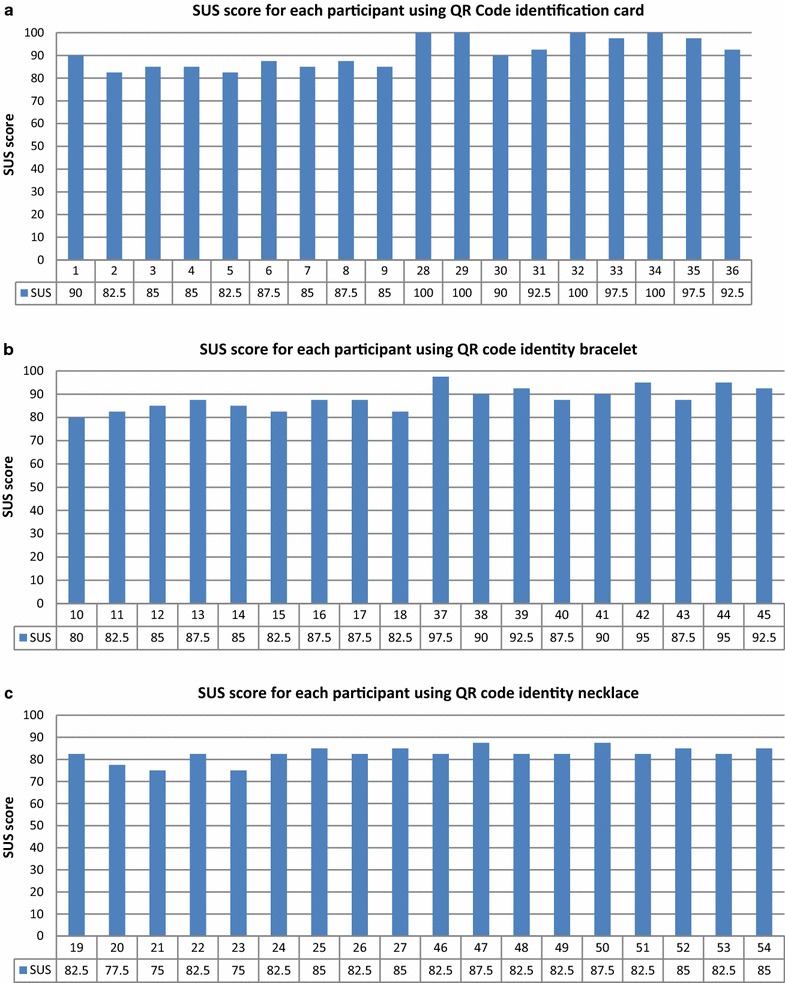
SUS score distribution for each participant who used **a** QR code identification card, **b** QR code identity bracelet, and **c** QR code identity necklace

Next, we compare performance by type of QR Code Identity Tag used. Figure 13 divides the participants’ SUS scores according to the assigned QR Code Identity Tag type.

**Fig. 14:**
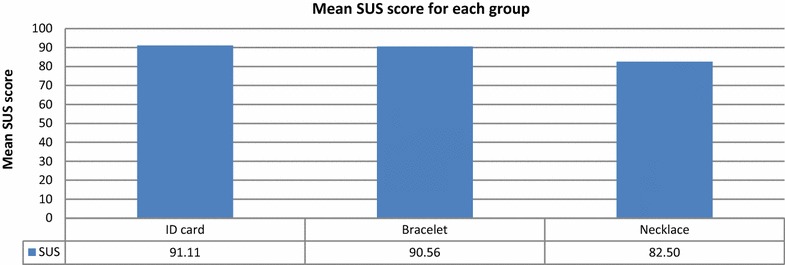
Mean SUS scores for groups with QR Code Identity Tags placed on identification cards, bracelets, and necklaces

Figure 14 shows the mean SUS scores for the groups with QR codes placed on identification cards, bracelets, and necklaces. The group using identification cards had the highest mean SUS score (91.11), as those participants did not feel any discomfort. The SUS score for the bracelet group was lower (90.56), as they found the system slightly cumbersome to use. The lowest score was obtained by the group using QR code necklaces (82.50), as some found the system cumbersome to use, and some would not like to use the system frequently. Although some participants felt slight discomfort, all groups’ mean SUS scores were above the success threshold.

### Group B

The parents of 60 children allowed their children to participate; the children were aged 5–16 years. Of the participants, 30 were asked to wear QR code identity bracelets, and 30 wore necklaces for 1 week. There were 20 children each in the age ranges of 5–8, 9–12, and 13–16 years.

Parents were requested to perform the following tasks:

1. Register on the QR Code Identity Tag website
2. Enter detailed information about the child on the QR Code Identity Tag website
3. Update detailed information about the child on the QR Code Identity Tag website
4. Scan the QR code.

Children were requested to perform the following task:

1. Wear the QR code identity necklace/bracelet

**Fig. 15:**
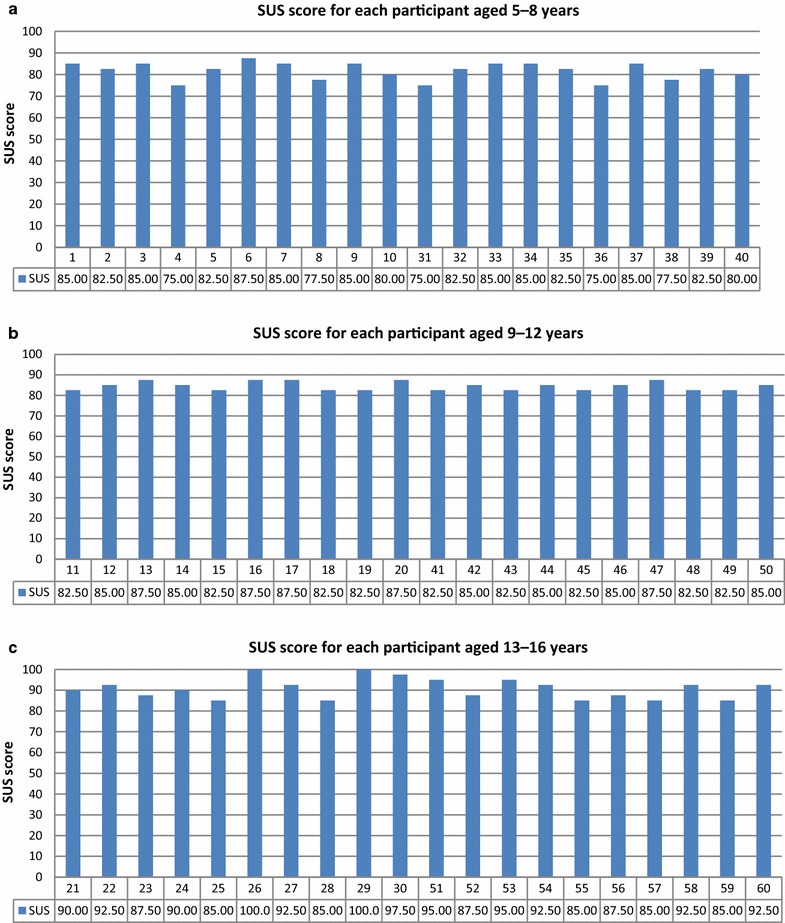
SUS score distribution for each participant according to age group: ages **a** 5–8 years, **b** 9–12 years, and **c** 13–16 years

After 1 week of using the system, parents completed the SUS questionnaire using feedback from their children. The SUS score distributions for each participant are presented according to age group in Fig. 15.

**Fig. 16:**
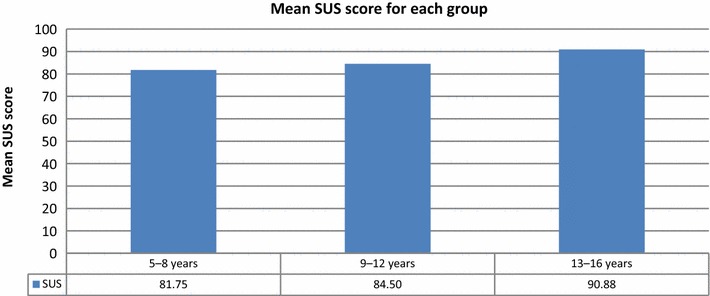
Mean SUS scores for the groups of children of various ages

The mean SUS scores for ages 5–8, 9–12, and 13–16 years were 81.75, 84.50, and 90.88, respectively (Fig. 16). Younger participants were less comfortable wearing the bracelets and necklaces than older participants were.

**Fig. 17:**
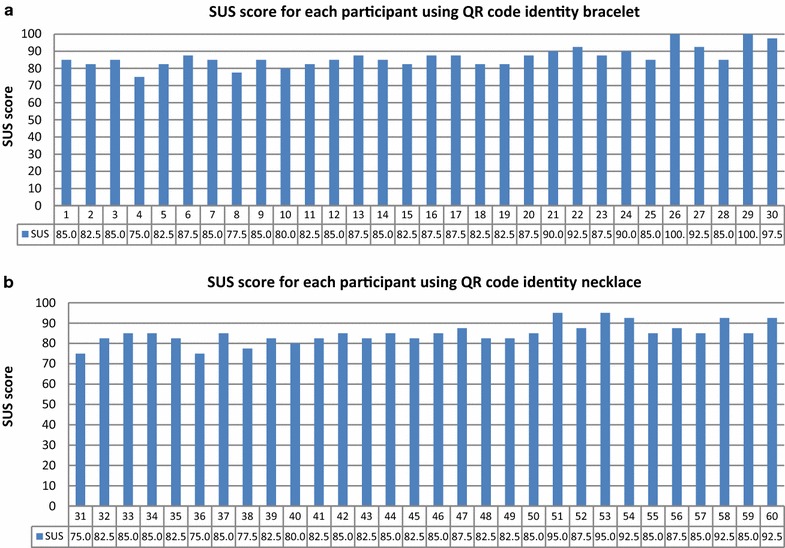
SUS score distribution for each participant using **a** QR code identity bracelet and **b** QR code identity necklace

Figure 17 depicts the SUS score of each participant according to the assigned QR Code Identity Tag type.

**Fig. 18:**
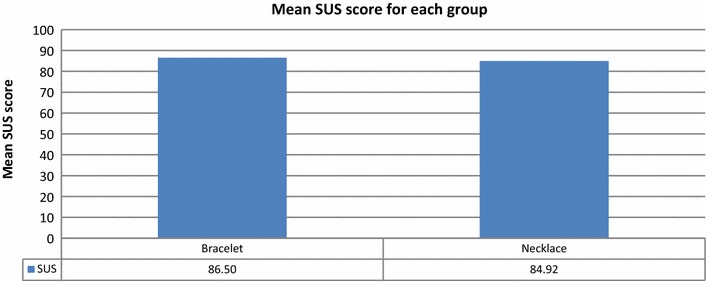
Mean SUS scores for groups with QR Code Identity tag placed on bracelet and necklace

**Fig. 19:**
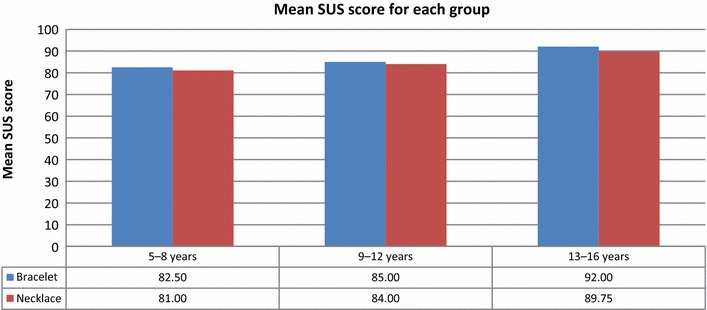
Mean SUS scores for various age groups and assigned identity tags

Figures 18 and 19 show that children of all age groups were more comfortable with the bracelet than the necklace. The average SUS score for all children assigned bracelets was 86.50, while that for children assigned necklaces was 84.92 (Fig. 18).

### Group C

This group consisted of 60 workers from 10 hospitals located in Istanbul; six workers from each hospital participated in the trial. Each hospital was required to have at least one computer, one Android smartphone, and an internet connection, and the participants were requested to download and install the QR Code Identity Tag mobile application. All the operations and steps were explained to all participants in the study. Each participant was requested to use all facilities of the QR Code Identity Tag system for 1 week.

They were requested to perform the following tasks:

1. Register patients
2. Create a unique online profile, password, identification number, and QR code for each patient
3. Take a detailed medical history from each patient
4. Create several copies of the QR code
5. Place one QR code copy on the door of the patient’s room, one on the bed, and another on the chart
6. Place a QR code bracelet on the patient’s wrist
7. Ensure that the patient places another QR code bracelet on the other wrist
8. Scan the QR codes on both bracelets to ensure that they belong to the same person
9. Scan the QR codes whenever patient identification is required.

We evaluated the system’s performance according to the participants’ computer skills.

We created the following groups:

- Group 1: No Computer Skills
- Group 2: Fundamental Skills (Typing, Mouse)
- Group 3: Basic Computing and Applications
- Group 4: Intermediate Computing and Applications
- Group 5: Advanced Computing and Applications
- Group 6: Proficient Computing, Applications, and Programming.

**Fig. 20:**
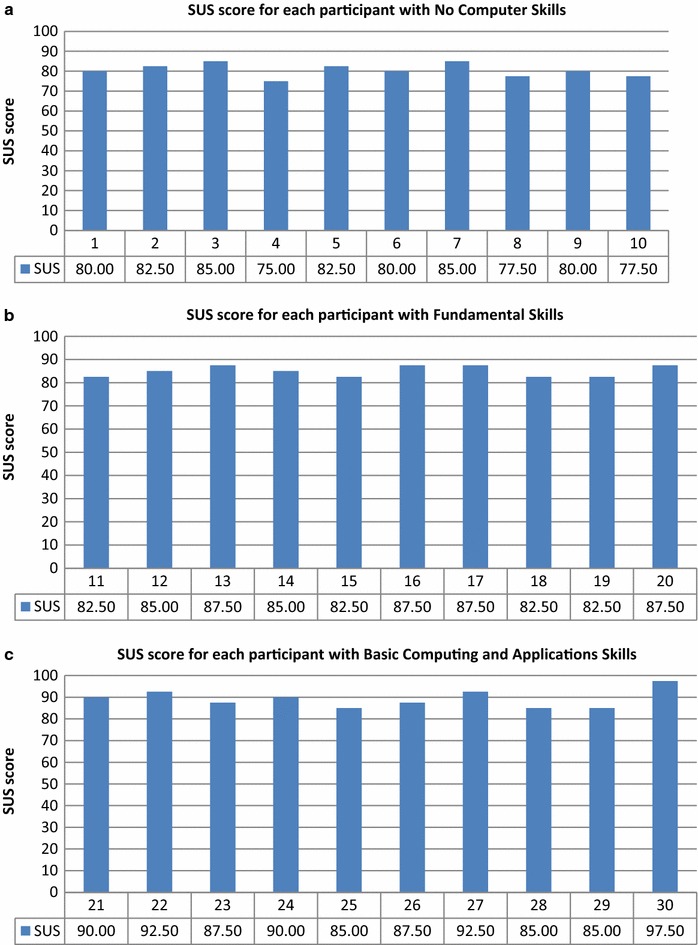
SUS score distributions for groups with various levels of computer skills. **a** No computer skills, **b** fundamental skills, and **c** basic computing and applications

**Fig. 21:**
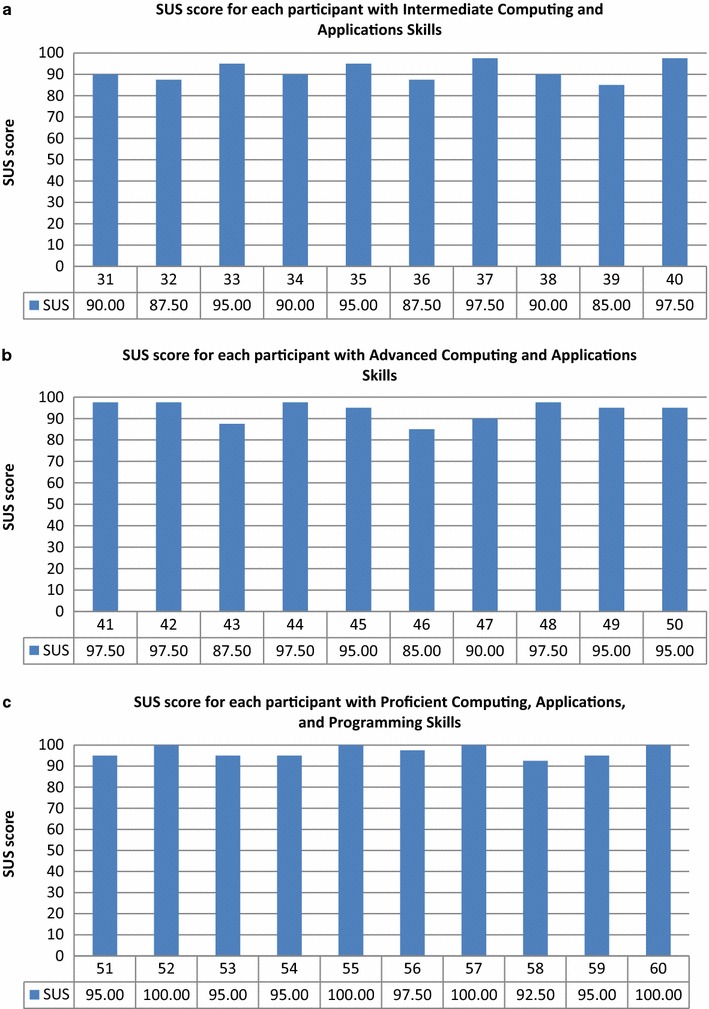
SUS score distributions for groups with various levels of computer skills. **a** Intermediate computing and applications, **b** advanced computing and applications, and **c** proficient computing, applications, and programming

The SUS score distributions for each participant at the various computer skill levels are presented in Figs. 20, 21.

**Fig. 22:**
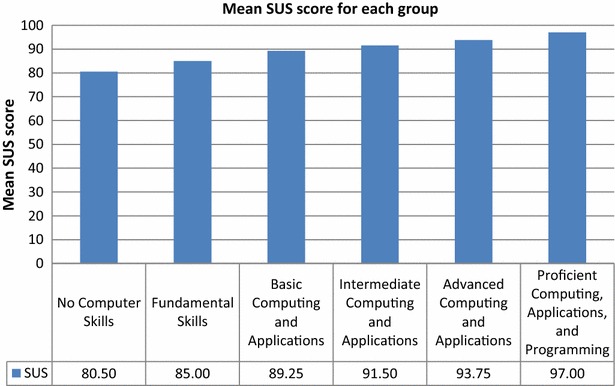
Mean SUS scores for computer skill level groups

Mean SUS score value was positively associated with computer skill level. The mean SUS score for the group with No Computer Skills (80.50) was the lowest, and that for the group with Proficient Computing, Applications, and Programming skills (97.00) was the highest, as expected (Fig. 22). Because the first group had no computer experience, they needed technical support to use the system, and they needed to learn things before they could get going with the system. In contrast, the participants with more computer experience felt competent using the system. Although the participants with no computer skills had some difficulties using the system, all groups’ mean SUS scores were above the success threshold.

### Comparison

**Fig. 23:**
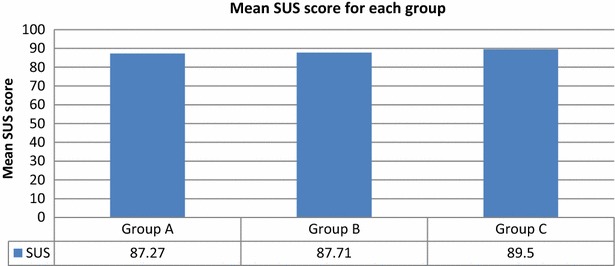
Mean SUS scores for each group

The mean SUS scores for Groups A, B, and C were 87.27, 87.71, and 89.50, respectively (Fig. 23). Each group had a mean SUS score above the success threshold, as was the overall mean score for all groups (88.16); thus, the developed system was usable and satisfactory.

**Fig. 24:**
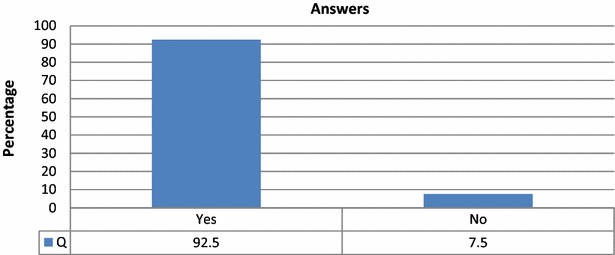
Answers to question about integration of the QR Code Identity Tag system into the Turkish healthcare system

Of the 174 total volunteers who participated in the study, 161 (92.5 %) voted to integrate the QR Code Identity Tag system into the Turkish healthcare system, while 13 (7.5 %) voted against it (Fig. 24).

## Conclusion

To decrease public healthcare costs and improve the quality of public healthcare, it is important to decrease medication errors, improve patient safety, and increase the accuracy of clinical procedures. Automatic patient authentication systems can positively affect these factors and enhance access to and delivery of public healthcare services.

We have implemented a QR Code Identity Tag system for Turkish healthcare. The QR Code Identity Tag offers smooth access to vital medical information. Once the QR code is read, general patient information, including name, address, and emergency contact, are displayed.

We chose QR codes because they are the most practical, cost-efficient alternative method of automation of patient authentication capabilities in public healthcare facilities with limited budgets.

The system does have some shortcomings. First, the mobile application works only on Android smartphones. In the near future, applications for other operating systems will be developed. Moreover, the system is currently available only in Turkish, the official language of Turkey. In the future, it will be extended to support several other languages spoken in Turkey: Kurdish, Albanian, Neo-Aramaic, Laz, Georgian, Bosnian, Bulgarian, Greek, Zazaki, Arabic, Azerbaijani, Kabardian, Armenian, Ladino, and Circassian. When new languages are supported, new usability evaluations will be performed using the SUS questionnaire translated into those languages.

Finally, according to the World Bank’s classification system, Turkey is an upper-middle income country (per-capita GDP: 10,970 dollars). However, Turkey ranks last in life expectancy at birth for women among the 34 OECD countries (see footnote 2; OECD Health Statistics 2014). The literacy rate is 91 % for women and 98.3 % for men. In 2013, Turkey had the third-highest level of income inequality among OECD member states (in terms of the Gini coefficient for income distribution) ((see footnote 2). Turkey also has significant regional inequalities, including those related to demographic factors, employment rates, and levels of education, infrastructure, welfare, and economic structure (Celebioglu and Dall’erba 2010; Çelebioğlu 2015; Aktas 2015; Ergin and Kunst 2015; Sayilan and Yildiz 2009). There are divisions between the eastern and western regions of Turkey in terms of agricultural and industrial development, working conditions, income level, the potential for public and private investment, and the direction of migration flow. Despite the instantiation of the Priority Provinces in Development program, there remains significant income inequality in the eastern region. For example, women’s illiteracy rate is reported to be maximal in the southeastern region (34.08 %), while it is only 6.30 % in the Marmara region. In the eastern and western regions, 64 and 35 % of the populations have no social security, respectively (Celebioglu and Dall’erba 2010; Çelebioğlu 2015; Gunay Aktas 2015; Ergin and Kunst 2015; Sayilan and Yildiz 2009). Furthermore, the availability, capacity, and quality of public services significantly differ between the eastern and western regions. These combined differences result in a highly unequal distribution of opportunities and living circumstances. In both the eastern and western regions, rural areas differ significantly from urban areas in terms of the same attributes listed above. Because the rural municipalities have limited financial sources, their populations are in decline, resulting in problems with service provision (Celebioglu and Dall’erba 2010; Çelebioğlu 2015; Gunay Aktas 2015; Ergin and Kunst 2015; Sayilan and Yildiz 2009). Therefore, it will be very challenging to implement the QR Code Identity Tag system in the rural areas and eastern regions of Turkey. The illiterate population will not be able to use the system at all. This system will be more easily implemented in the urban areas and western parts of Turkey, where the majority of inhabitants are literate, have higher incomes, own smartphones, have internet connections, and have computers at home.

We evaluated the performance of the proposed system using the SUS, which has been shown to distinguish effectively between unusable and usable systems as well as or better than proprietary questionnaires. The mean overall SUS score for the QR Code Identity Tag system (88.16) was above the success threshold; thus, users were satisfied with the product and likely to recommend it to their friends. Of the 174 volunteers from different areas of Istanbul who were able to use the system effectively, 161 (92.5 %) voted to integrate the QR Code Identity Tag system into the Turkish healthcare system. Consequently, we believe that the QR Code Identity Tag service should be integrated with the currently evolving Turkish healthcare system. It will help to prevent medical errors that occur during emergency situations and significantly improve public satisfaction with Turkish healthcare services.

## Footnotes

1 http://www.ccforpatientsafety.org/common/pdfs/ICPS/Patientsolutions.pdf.

2 OECD Statistics https://stats.oecd.org/.

3 HealthVault https://www.healthvault.com.

4 PatientsLikeMe https://www.patientslikeme.com/.

5 childIDcode http://www.childidcode.com/.

6 ICE ID http://www.iceid.co.uk/.

7 ENDEVR http://www.endevr.com/.

8 If I Need Help https://ifineedhelp.org/.

9 Keep Me Safe ID http://keepmesafeid.com.

10 LIFETAG http://lifetag.com/.

11 TAGGISAR http://www.taggisar.co.uk/.

12 Elegant Medical Alert http://www.elegantmedicalalert.com/.

13 Brenmoor http://brenmoor.com/.

14 Adobe https://www.adobe.com.

15 MySQL https://www.mysql.com/.

16 Android https://www.android.com.

17 Eclipse https://eclipse.org/.

18 Android software development kit https://developer.android.com/studio/index.html.

19 IntelliJ IDEA IDE https://www.jetbrains.com/idea/.
